# Low-fat dietary pattern and dietary advanced glycation end-products intake: a secondary analysis of the Women’s Health Initiative randomized trial

**DOI:** 10.1038/s41416-026-03514-x

**Published:** 2026-07-28

**Authors:** Margaret S. Pichardo, Rebecca P. Hunt, Lindsay L. Peterson, Kathy Pan, Mace Coday, Su Yon Jung, Linda Snetselaar, Marian L. Neuhouser, Susan E. Steck, Fred K. Tabung, Nazmus Saquib, JoAnn E. Manson, Rowan T. Chlebowski

**Affiliations:** 1 Department of Surgery, Penn Medicine, Philadelphia, PA, USA.; 2 Public Health Sciences Division, Fred Hutchinson Cancer Center, Seattle, WA, USA.; 3 Division of Medical Oncology, Department of Medicine, Washington University, Siteman Cancer Center, St. Louis, MO, USA.; 4 Kaiser Permanente Southern California, Downey, CA, USA.; 5 Department of Preventive Medicine, University of Tennessee Health Science Center, Memphis, TN, USA.; 6 Translational Sciences Section, School of Nursing, Department of Epidemiology, Fielding School of Public Health, Jonsson Comprehensive Cancer Center, University of California, Los Angeles, CA, USA.; 7 Epidemiology and Endocrinology/Metabolism, University of Iowa, Iowa City, IA, USA.; 8 Department of Epidemiology & Biostatistics, Arnold School of Public Health, University of South Carolina, Columbia, SC, USA.; 9 Division of Medical Oncology, Department of Internal Medicine, The Ohio State University College of Medicine and Comprehensive Cancer Center, Columbus, OH, USA.; 10 Clinical Sciences Department, College of Medicine, Sulaiman AlRajhi University, Al-Qassim, Saudi Arabia.; 11 Department of Medicine, Brigham and Women’s Hospital, Harvard Medical School, Boston, MA, USA.; 12 The Lundquist Institute, Torrance, CA, USA.

## Abstract

**BACKGROUND:**

In the Women’s Health Initiative (WHI) Dietary Modification (DM) randomized trial, a low-fat dietary pattern intervention reduced breast cancer mortality (*P* = 0.02). Higher dietary advanced glycation end-products (dAGE) may be associated with higher breast cancer incidence. In this report, we examined whether the WHI dietary intervention influenced dAGE consumption.

**METHODS:**

Of 48,835 postmenopausal women randomized (40:60) to dietary intervention versus usual diet comparison, 40,209 had food frequency questionnaires at baseline, and serially through 5–7 years, which were used to estimate dAGE scores (kilo Unit/1000 kilocalories [kU/1000 kcal]) using a commonly referenced database. Multivariate regressions with repeated dAGE measures were examined by randomization group.

**RESULTS:**

Baseline mean dAGE scores were similar for intervention (7542 kU/1000 kcal, standard deviation (SD) = 912) and comparison (7514 kU/1000 kcal, SD = 917) groups. Through 5–7 years, dAGE scores were persistently lower in intervention versus comparison groups (mean range 5361–6236 kU/1000 kcal versus 7159–7669 kU/1000 kcal (*P* < 0.0001).

**CONCLUSIONS:**

The WHI low-fat dietary pattern intervention substantially reduced dAGE consumption.

**CLINICAL TRIAL REGISTRATION:**

NCT00000611; Date: 10/28/1999.

## INTRODUCTION

In the Women’s Health Initiative (WHI) Dietary Modification (DM) randomized trial involving 48,835 postmenopausal women, a low-fat dietary pattern intervention compared with a usual diet, reduced fat intake and increased consumption of fruits, vegetables and grains [1]. Over 20 years of follow-up, the intervention was associated with a statistically significant early and sustained reduction in the incidence of estrogen receptor-positive, progesterone receptor-negative, breast cancers (HR, 0.77; 95% CI, 0.64–0.94) as well as a reduction in breast cancer mortality (hazard ratio [HR] 0.79, 95% confidence interval [CI] 0.64–0.97, *P* = 0.02) [2].

Efforts to understand the mechanisms underlying this mortality benefit have focused in part on metabolic pathways. The WHI dietary intervention was shown to reduce the number of metabolic syndrome components, including central obesity, hypertension, dyslipidemia, and diabetes history [3], leading to the hypothesis that metabolic health may influence breast cancer outcomes. Supporting this, Pan et al. reported that among women in the intervention group, those with three or four components at baseline experienced a 69% reduction in breast cancer mortality compared with those with no components (HR 0.31 95% CI 0.14–0.69, interaction *P* = 0.01) [4].

Advanced glycation end products (AGEs) are reactive metabolites formed endogenously and consumed through the diet, particularly in foods high in fat and processing [5]. Dietary AGEs have been implicated in metabolic dysfunction [6], obesity [5], chronic inflammation [7, 8] and cancer [9-11], including higher breast cancer incidence [12, 13]. Experimental and observational evidence indicate that foods of animal origin, particularly red and processed meats, high-fat spreads and baked or fried foods, are among the highest contributors to dietary AGE intake, especially when prepared using dry heat methods, whereas fruits, vegetables, whole grains and legumes are generally lower in AGE content [5].

Given that the WHI dietary intervention reduced intake of foods expected to be high in AGE and increased intake of foods expected to be lower in AGE, we examined whether a low-fat dietary intervention, compared with a usual diet, was associated with changes in dietary AGE (dAGE) intake over time in a large randomized clinical trial.

## MATERIALS AND METHODS

The WHI DM trial design and conduct have been described [14, 15], 48,835 postmenopausal women, aged 50–79 years, were enrolled at 40 US clinical centers from 1993 through 1998 and followed for a primary endpoint of incidence of breast cancer and colorectal cancer. The study was approved at participating clinical centers and all participants gave written informed consent. The randomization algorithm was implemented by the WHI Clinical Coordinating Center (Seattle, WA) and assigned women in a 40:60 ratio to a low-fat dietary pattern (*n* = 19,541) or usual diet comparison (*n* = 29,294) group. The Trial Registration # NCT00000611 [16]. This report follows the Consolidated Standard of Reporting Trials (CONSORT).

Baseline characteristics were collected through interviews for medication use and by self-report questionnaires for other variables. Dietary intake was monitored using food frequency questionnaires at baseline, 1-year, and thereafter annually in a rotating subgroup of about a third of participants throughout the 7-year intervention period. Annual measurements were obtained for body weight and height, with body mass index (BMI) calculated by blinded trained clinical staff [15].

The dietary intervention goal was to reduce fat intake to 20% of total energy and increase consumption of fruits, vegetables, and grains. Intervention goals did not include caloric restriction or weight loss. The dietary intervention involved 18 group sessions led by centrally trained registered dietitians and nutritionists in year one and quarterly maintenance sessions that lasted through the intervention period. Comparison group women received written health-related materials only.

In a previous report, the N^Ɛ^-carboxymethyl-lysine (CML)-AGE content of 540 selected foods was determined using an enzyme-linked immunosorbent assay (ELISA) based on monoclonal anti-CML antibody (detailed in Uribarri 2010 [5]). The CML-AGE values from the database of Uribarri et al. were matched to each food item on food frequency questionnaires (FFQ) [17] and used as a dAGE measure in studies evaluating dAGE intake [12, 13, 18]. Adopting this method, WHI investigators at the Fred Hutchinson Cancer Center matched CML-AGE values to food items on the WHI FFQ and, in analyses adjusted for total energy density using nutrient density methods [19], estimated dAGE values in WHI participants in a published report [20]. In the current analyses, total dAGE was used as a continuous measure and categorized into quintiles.

Included in the current analyses were 40,209 DM participants with baseline and annual follow-up food frequency questionnaire data. Baseline demographic, lifestyle and dietary characteristics were examined by DM trial randomization group. *P*-values for comparison across groups were computed from Chi-square tests of independence for categorical variables and two-sample t-tests for continuous variables. Tests for dietary variables were conducted on the log scale.

A generalized linear model with repeated measures was used to assess the effect of DM randomization on dAGE (between subjects), study year (within subjects) and the DM randomization group * study year interaction. As dietary monitoring was done in a 1/3 rotating sample after year 1, visit years 2–4 and 5–7 are grouped to ensure consistency in comparisons across time points. Building on this, additional generalized linear models were developed to examine the effect of the DM low-fat dietary pattern intervention versus usual diet comparison on dAGE by randomization group with analysis variables identified through literature review [20], over time using repeated measures on dAGE. *P*-values for varying effects on dAGE by trial randomization group, by visit and by trial randomization group and visit with each analysis variable are displayed. Least squares means for dAGE are plotted over time (by study year) and DM trial arm. All *p*-values are two-sided with a significance level of 0.05. Fifty-four tests were performed; approximately three tests would be expected to be significant by chance alone. All analyses were performed using SAS for Windows, version 9.4 (SAS Institute Inc., Cary, NC).

## RESULTS

The analytical sample of 40,209 included 16,198 women randomized to the intervention and 24,011 randomized to the comparison group. At baseline, both randomization groups had similar demographics, medical history, and lifestyle factors. The mean (standard deviation (SD)) dAGE scores were also similar: intervention: 7542 kU/1000 kcal (SD = 912); comparison: 7514 kU/1000 kcal (SD = 917) (Table 1).

**Fig. 1:**
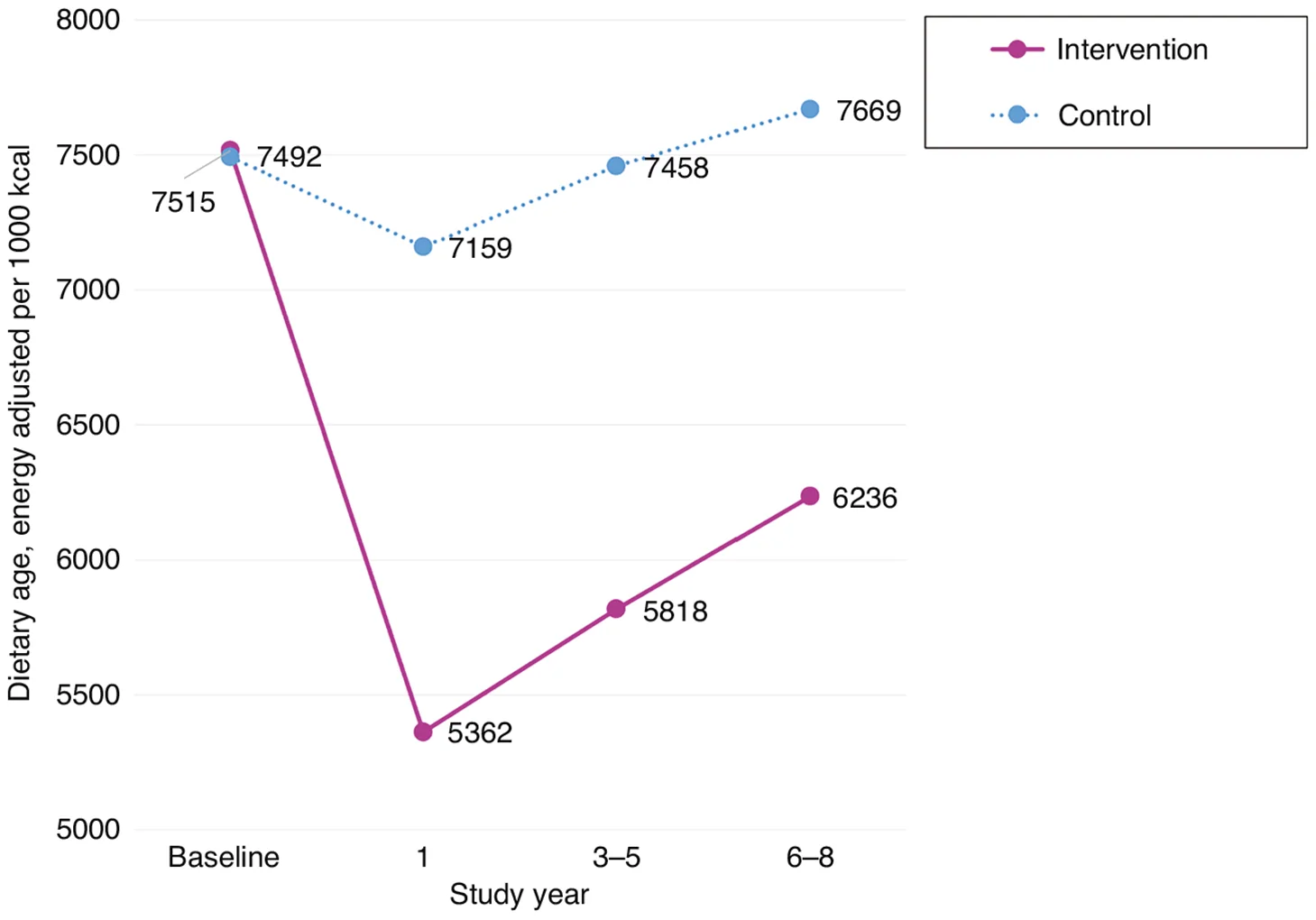
Dietary Advanced Glycation End Products (AGE) least squares means* by dietary modification trial arm and study visit year at follow-up. Standard errors for Intervention/Control at each Study Year are 23/18, 24/20, 26/21, 29/23 at baseline, year 1, years 3–5 and years 6–8, respectively.

Dietary AGE least squares means by DM trial randomization group and follow-up year are shown in Fig. 1. The largest difference in dAGE was observed after 1-year follow-up (intervention: 5362 kU/1000 kcal versus comparison: 7159 kU/1000 kcal). Subsequently, persistently lower dAGE levels in the intervention versus comparison group women, with some attenuation of the difference: visit year 2–4 and visit year 5–7 (Fig. 1). Our findings were robust to inclusion of covariates in multivariable regression with repeated measures (Table 2; beta coefficients (*p*-value): baseline 23.53 (*P* = 0.43), year 1 (−1796.96 kU/1000 kcal, *P* < 0.0001), year 3–5 (−1639.87, *P* < 0.0001), years 6–8 (−1,432.46, *P* < 0.0001); between subjects, DM trial arm, *F-statistic* 2312.17, *P* < 0.0001; Within subjects, study visit, *F-statistic* 1845.50, *P* < 0.0001; Interaction DM trial arm * study visit, *F-statistic* 1168.89, *P* < 0.0001).

Descriptive statistics for the baseline sample by trial arm are shown in Supplementary Table 1. Effect modification by covariates were examined and shown in Supplementary Table 2 and Supplementary Figs. 1-3. Overall, a positive effect modification was detected for Age at screening (*F-statistic* 4.90, *P* < 0.0001), Family income (*F-statistic* 2.33, *P* = 0.03), Smoking status (*F-statistic* 2.77, *P* = 0.01), body mass index (*F-statistic* 4.67, *P* < 0.0001), Fruits and vegetables (*F-statistic* 3.06, *P* = 0.001), and total HEI-2015 score quartiles (*F-statistic* 5.15, *P* < 0.0001).

## DISCUSSION

In the current analysis, a low-fat dietary pattern was associated with reductions in dAGE intake over 7 years of follow-up. These findings build on prior WHI evidence linking dAGE exposure and dietary patterns to breast cancer outcomes. Among 2023 women with breast cancer enrolled in the WHI Observational Study and the WHI DM trial, higher dAGE intake has been associated with increased risk of breast cancer-specific, cardiovascular, and all-cause mortality [20]. Similarly, a secondary analysis of the WHI DM randomized trial among 1764 women diagnosed with breast cancer demonstrated improved 10-year breast cancer survival in the dietary intervention versus the usual-diet comparison group (82% vs 78%, *P* = 0.01) [21].

The present report extends these observations by demonstrating that the WHI low-fat dietary intervention was associated with a significant and sustained reduction in dAGE intake. Although the intervention was not specifically designed to reduce dAGE intake, it produced meaningful changes in dietary patterns relevant to dAGE exposure. Early in the intervention, total fat intake decreased by 24.3 g/day relative to controls, driven primarily by reductions in added fats (−9.1 g/day), meats (−4.6 g/day), and desserts (−3.9 g/day) [22]. These changes reflect reduced consumption of foods such as butter, oils, dressings, red and processed meats, and baked goods, which are recognized contributors to dietary AGE intake. Over a longer follow-up, these changes were partially attenuated but remained substantial, with fat intake reduced to approximately 25% of energy at year 1 and ~30% at years 5–7 compared with ~35–36% in controls, alongside increased intake of fruits, vegetables, and grains [23].

These dietary shifts are consistent with patterns expected to lower dAGE exposure, as diets higher in animal fats and processed foods tend to be AGE-rich, whereas plant-based foods are generally lower in dAGE. The WHI low-fat dietary pattern also shares features with the Dietary Approaches to Stop Hypertension (DASH) diet, characterized by higher intake of fruits, vegetables, and grains and lower intake of red meat and higher-fat foods [24]. Furthermore, evidence from the Prostate, Lung, Colorectal, and Ovarian (PLCO) cohort supports the relevance of these dietary changes to dAGE exposure [12]. In PLCO, dAGE intake was primarily driven by fats and oils (~21%), red meat (~15%), mixed dishes (~15%), and processed meats (~9%), whereas fruits and vegetables contributed minimally (<3%) [12]. These patterns align closely with the WHI intervention, which reduced intake of fats and meats while increasing plant-based foods, supporting the plausibility of the observed reductions in dAGE. Together, these findings suggest that adoption of a low-fat, plant-forward dietary pattern may contribute to meaningful reductions in dAGE intake in free-living populations.

Importantly, the WHI DM intervention did not explicitly target dAGE reduction through mechanisms known to influence AGE exposure, such as modification of cooking methods, reduction of ultra-processed foods, or specific food preparation practices. This represents a key limitation, as the relative contribution of these factors to the observed reduction in dAGE cannot be determined. Notably, emerging interventional studies are now directly targeting dAGE reduction, including a recently completed clinical trial in breast cancer survivors, with results pending, evaluating the effects of a low-AGE diet on metabolic and inflammatory biomarkers and potential prognostic pathways (NCT05265715). Additionally, dAGE estimates were derived from FFQ data and are not directly linkable to individual foods or preparation methods, limiting mechanistic interpretation. Importantly, the reduction in dAGE observed in this study occurred without targeted modification of cooking methods or food processing, that broader dietary pattern changes alone may substantially influence dAGE exposure. Nonetheless, this analysis provides novel empirical evidence from a large randomized trial that a broadly implemented dietary pattern intervention can result in meaningful and sustained reductions in dAGE exposure.

Higher dAGE intake may influence insulin resistance, metabolic dysfunction [6, 25] and weight gain [26], factors associated with adverse breast cancer outcomes. In the WHI DM trial, a low-fat dietary pattern intervention was associated with reduced metabolic syndrome risk [3], and higher metabolic syndrome burden has been linked to poorer prognosis breast cancers (*p* = 0.03) and increased breast cancer-specific mortality (HR 1.44, 95% CI 1.02–2.04, *p* < 0.03) [4]. These pathways may provide a biologically plausible link between dietary patterns, dAGE exposure, and breast cancer outcomes, although causal mediation was not evaluated in the present study.

Strengths of this study include the randomized trial design, a large and diverse cohort of 40,209 post-menopausal women, and repeated dietary assessments over 7 years. Limitations include the secondary, hypothesis-generating nature of the analysis, reliance on self-reported dietary intake, and the inability to directly link dAGE estimates to specific foods or preparation methods.

## CONCLUSION

Among postmenopausal women, a low-fat dietary pattern intervention resulted in a sustained reduction in dAGE intake versus usual diet comparison. These findings provide randomized clinical trial evidence that the WHI low-fat dietary pattern may represent an effective strategy to reduce dietary AGE exposure. Future studies will explore whether reduced dAGE intake associated with the low-fat eating pattern contributes to favorable breast cancer outcomes in the WHI DM trial.

## Supplementary Material

Supplemental Material

Supplementary information The online version contains supplementary material available at https://doi.org/10.1038/s41416-026-03514-x.

## Figures and Tables

**Table 1.** Demographic, lifestyle and dietary AGE intake characteristics.

	DM Trial Arm				*P*-value^a^
	Intervention		Control		
	*N*	Mean (SD) or %	*N*	Mean (SD) or %	
Demographics					
Age at screening, y	16,198	62.3 (6.8)	24,011	62.4 (6.8)	0.29
50–59	5858	36.2	8640	36.0	
60–69	7698	47.5	11,349	47.3	
70–79	2642	16.3	4022	16.8	
Race^b^					0.52
American Indian/Alaska Native	39	0.2	59	0.2	
Asian	148	0.9	215	0.9	
Native Hawaiian/Other PI	6	0.0	3	0.0	
Black	1601	9.9	2266	9.4	
White	14,025	86.6	20,921	87.1	
More than one race	180	1.1	262	1.1	
Other/not reported	199	1.2	285	1.2	
Ethnicity^c^					0.06
Not Hispanic/Latino	15,528	95.9	23,023	95.9	
Hispanic/Latino	621	3.8	943	3.9	
Not reported	49	0.3	45	0.2	
Education					0.16
<High school diploma/GED	622	3.9	1019	4.3	
High school diploma/GED	2857	17.7	4202	17.6	
School after high school	6293	39.1	9402	39.4	
College degree or higher	6335	39.3	9242	38.7	
Family income					0.21
<$20,000	2164	14.2	3327	14.7	
$20,000–$49,999	7077	46.3	10,523	46.5	
≤$50,000+	6049	39.6	8793	38.8	
Medical history					
Antihypertensive medication use					0.28
Calcium channel blocker (CCB)	1549	9.6	2227	9.3	
Non-CCB	3075	19.0	4693	19.5	
Non-user	11,574	71.5	17,091	71.2	
Unopposed estrogen use					0.94
Never	10,303	63.6	15,264	63.6	
Past	1840	11.4	2752	11.5	
Current	4045	25.0	5975	24.9	
Estrogen + progesterone use					0.44
Never	11,624	71.8	17,277	72.0	
Past	1426	8.8	2028	8.4	
Current	3144	19.4	4700	19.6	
Lifestyle Risk Factors					
Smoking status					0.87
Never	8332	52.0	12,426	52.2	
Past	6690	41.7	9866	41.5	
Current	1003	6.3	1495	6.3	
Body mass index, kg/m^2^	16,132	29.1 (5.9)	23,900	29.0 (5.8)	0.23
<25	4259	26.4	6353	26.6	
25-<30	5794	35.9	8608	36.0	
≥30	6079	37.7	8939	37.4	
Episodes per week of moderate to vigorous recreational physical activity					
None	6490	40.1	9731	40.5	0.66
Any, median (IQR)	8035	3.0 (2.0–6.0)	11,900	3.0 (2.0–6.0)	
Alcohol intake					
Non-drinker	6555	40.5	9731	40.5	0.92
Any, median (IQR) drinks/day	9637	0.2 (0.1–0.7)	14,278	0.2 (0.1–0.7)	
Diet					
Total energy, kcal/day	16,198	1802 (689)	24,011	1801 (680)	0.85
Fruits and vegetables, servings/day	16,198	2.9 (1.3)	24,011	2.9 (1.3)	0.64
Whole grains, servings/day	16,198	1.1 (0.9)	24,011	1.1 (0.9)	0.61
Red and processed meat, servings/day	16,198	2.4 (1.7)	24,011	2.4 (1.7)	0.31
Total HEI-2005 score	16,198	63.9 (9.9)	24,011	63.9 (10.0)	0.89
Total HEI-2015 score	16,198	61.6 (9.4)	24,011	61.6 (9.5)	0.83
DASH diet score	16,198	24.4 (4.4)	24,011	24.3 (4.5)	0.39
9–20	3202	19.8	4888	20.4	
21–23	3520	21.7	5279	22.0	
24–25	2767	17.1	3966	16.5	
26–28	3723	23.0	5535	23.1	
29–38	2986	18.4	4343	18.1	
Dietary AGE quintiles, kU/1000 kcal	16,198	7542 (912)	24,011	7514 (917)	0.15
≤5974	3212	19.8	4830	20.1	
5974–6876	3207	19.8	4834	20.1	
6877–7763	3196	19.7	4847	20.2	
7764–8922	3299	20.4	4743	19.8	
>8922	3284	20.3	4757	19.8	

*AGE* advanced glycation end product, *DM* dietary modification, *IQR* interquartile range, *SD* standard deviation.

a *P*-values are from Chi-square tests of independence for categorical variables and 2-sample t-tests for continuous variables. Two sample t-tests for dietary variables were conducted on the log scale.

b Race and ethnic group were self-reported by participants. Multiracial participants self-identified with >1 race. Participants of other races self-identified with that category.

**Table 2.** Multivariate regression with repeated measures for dietary AGE (kU/1000 kcal) by DM trial arm and study visit year at follow-up.

Main Effects	F-statistic	*p*-value
Between subjects (DM trial arm)	2312.17	<0.0001
Within subjects (visit)	190.19	<0.0001
Interaction (DM trial arm * visit)	1168.89	<0.0001
Parameter Estimates for DM trial arm by study year	*β* coefficient	*p*-value
Baseline	23.53	0.43
Year 1	−1796.96	<0.0001
Years 3–5	−1639.87	<0.0001
Years 6–8	−1432.46	<0.0001

## Data Availability

Access to the WHI data may be obtained by visiting the following website: https://www.whi.org/propose-a-paper.
