# Supplementary material for: Low-fat dietary pattern and dietary advanced glycation end-products intake: a secondary analysis of the Women’s Health Initiative randomized trial
Source: Br J Cancer. 2026 Jul 28;135(9):1424–9. doi: 10.1038/s41416-026-03514-x (PMC13564117; doi:10.1038/s41416-026-03514-x)

Low-Fat Dietary Pattern and Dietary Advanced Glycation End-Products intake: A Secondary Analysis of The Women's Health Initiative Randomized Trial

Authors:

Margaret S. Pichardo, MD, PhD, MPH[1*], Rebecca P. Hunt, M.S.[2], Lindsay L. Peterson, MD, MSCR[3], Kathy Pan, MD[4], Mace Coday, PhD[5], Su Yon Jung PhD, MPH[6], Linda Snetselaar, PhD, RDN, FAND, LD[7], Marian L Neuhouser, PhD, RD[2], Susan E. Steck PhD, MPH, RDN, FAND[8], Fred K. Tabung, PhD, MSPH[9], Nazmus Saquib, MBBS, MBA, MPH, PhD[10], JoAnn E. Manson, MD, DRPH[11], Rowan T. Chlebowski, MD, PhD[12]

1 Department of Surgery, Penn Medicine, Philadelphia, PA, USA
2 Public Health Sciences Division, Fred Hutchinson Cancer Center, Seattle, WA, USA
3 Division of Medical Oncology, Department of Medicine, Washington University, Siteman Cancer Center, St. Louis MO, USA
4 Kaiser Permanente Southern California, Downey, CA, USA
5 Department of Preventive Medicine, University of Tennessee Health Science Center, Memphis, TN, USA
6 Translational Sciences Section, School of Nursing, Department of Epidemiology, Fielding School of Public Health, Jonsson Comprehensive Cancer Center, University of California, Los Angeles, California, USA
7 Epidemiology and Endocrinology/Metabolism, University of Iowa, Iowa, USA
8 Department of Epidemiology & Biostatistics, Arnold School of Public Health, University of South Carolina, Columbia, SC, USA
9 Division of Medical Oncology, Department of Internal Medicine, The Ohio State University College of Medicine and Comprehensive Cancer Center, Columbus, Ohio, USA
10Clinical Sciences Department, College of Medicine, Sulaiman AlRajhi University, Al-Bukairyah, Al-Qassim, Saudi Arabia
11 Department of Medicine, Brigham and Women's Hospital, Harvard Medical School, Boston, MA, USA
12 The Lundquist Institute, Torrance, CA, USA

Corresponding Author:
*Margaret S Pichardo, MD, PhD, MPH**,** Department of Surgery, Penn Medicine, 3400 Spruce Street, Maloney 4, Philadelphia, PA 19104; Margaret.pichardo@pennmedicine.upenn.edu

Supplementary Tables

Supplementary Table 1. Mean (SD) dietary AGE (kU/1000 kcal) by DM trial arm and selected baseline characteristics

	DM Trial Arm			
	Intervention		Control	
	N	Mean (SD)	N	Mean (SD)
**Demographics**				
Age at screening, y				
50-59	5858	7751(942)	8640	7729(961)
60-69	7698	7527(903)	11349	7479(886)
70-79	2642	7119(793)	4022	7149(848)
Race[1]				
American Indian/Alaska Native	39	8285(491)	59	8258(595)
Asian	148	7067(706)	215	7360(895)
Native Hawaiian/Other PI	6	7448(50)	3	7122(740)
Black	1601	8391(446)	2266	8406(477)
White	14025	7442(811)	20921	7408(814)
More than one race	180	7752(929)	262	7919(971)
Other/Not reported	199	7738(135)	285	7788(70)
Ethnicity[1]				
Not Hispanic/Latino	15528	7523(896)	23023	7496(910)
Hispanic/Latino	621	8002(190)	943	7916(29)
Not reported	49	7473(322)	45	8178(928)
Education				
< High school diploma/GED	622	7779(285)	1019	7806(201)
High school diploma/GED	2857	7542(946)	4202	7474(923)
School after high school	6293	7588(920)	9402	7593(959)
College degree or higher	6335	7473(840)	9242	7415(830)
Family income				
<$20,000	2164	7580(106)	3327	7618(170)
$20,000-$49,999	7077	7531(899)	10523	7501(919)
≤$50,000+	6049	7559(858)	8793	7507(802)
**Lifestyle Risk Factors**				
Smoking status				
Never	8332	7444(881)	12426	7459(895)
Past	6690	7601(932)	9866	7515(913)
Current	1003	7957(940)	1495	7956(66)
Body mass index, kg/m^2				
<25	4259	7195(757)	6353	7161(746)
25 - <30	5794	7480(879)	8608	7439(834)
≥30	6079	7845(995)	8939	7839(56)
Episodes per week of moderate to vigorous recreational physical activity				
None	6490	7689(12)	9673	7648(993)
>0 - 2	2526	7564(894)	3845	7540(863)
>2 - 4.5	2676	7450(834)	3878	7422(824)
>4.5	2833	7354(831)	4177	7318(875)
Alcohol intake				
Non-drinker	6555	7546(67)	9731	7540(65)
<0.13	3585	7536(812)	5188	7467(890)
0.13 - <0.48	2825	7539(830)	4344	7467(789)
>= 0.48	3227	7538(757)	4746	7554(735)

[1] Race and ethnic group were self-reported by participants. Multiracial participants self-identified with >1 race. Participants of other races self-identified with that category.

AGE, Advanced Glycation End Product; DM, Dietary Modification

Supplementary Table 2. Multivariate regression with repeated measures for dietary AGE (kU/1000 kcal) by DM trial arm, study visit, and baseline characteristics

Effect Modification:	F-statistic	P-value
Age at screening, y		
Age at screening * DM trial arm	5.57	0.004
Age at screening * visit	8.77	<0.0001
Age at screening * DM trial arm * visit	4.90	<0.0001
Race		
Race * DM trial arm	0.87	0.52
Race * visit	2.55	0.0003
Race * DM trial arm * visit	1.30	0.18
Ethnicity		
Ethnicity * DM trial arm	0.56	0.57
Ethnicity * visit	0.84	0.54
Ethnicity * DM trial arm * visit	0.46	0.84
Education		
Education * DM trial arm	1.65	0.18
Education * visit	2.43	0.01
Education * DM trial arm * visit	1.02	0.42
Family income		
Family income * DM trial arm	1.83	0.16
Family income * visit	2.92	0.01
Family income * DM trial arm * visit	2.33	0.03
Antihypertensive medication use		
Antihypertensive medication use * DM trial arm	3.32	0.04
Antihypertensive medication use * visit	2.22	0.04
Antihypertensive medication use * DM trial arm * visit	1.13	0.34
Unopposed estrogen use		
Unopposed estrogen use * DM trial arm	0.82	0.44
Unopposed estrogen use * visit	0.66	0.68
Unopposed estrogen use * DM trial arm * visit	0.58	0.75
Estrogen + progesterone use		
Estrogen + progesterone use * DM trial arm	1.06	0.35
Estrogen + progesterone use * visit	0.76	0.60
Estrogen + progesterone use * DM trial arm * visit	1.98	0.06
Smoking status		
Smoking status * DM trial arm	3.43	0.03
Smoking status * visit	2.04	0.06
Smoking status * DM trial arm * visit	2.77	0.01
Body mass index, kg/m^2		
Body mass index * DM trial arm	2.74	0.06
Body mass index * visit	18.62	<0.0001
Body mass index * DM trial arm * visit	4.67	<0.0001
Episodes per week of moderate to vigorous recreational physical activity		
Episodes/wk moderate/vigorous physical activity * DM trial arm	0.93	0.42
Episodes/wk moderate/vigorous physical activity * visit	1.65	0.09
Episodes/wk moderate/vigorous physical activity * DM trial arm * visit	1.59	0.11
Alcohol intake, drinks/day		
Alcohol intake * DM trial arm	2.51	0.06
Alcohol intake * visit	1.36	0.20
Alcohol intake * DM trial arm * visit	1.45	0.16
Total energy quartiles, kcal/day		
Total energy * DM trial arm	1.22	0.30
Total energy * visit	1.28	0.24
Total energy * DM trial arm * visit	1.29	0.24
Fruit and vegetable quartiles, servings/day		
Fruits and vegetables * DM trial arm	2.96	0.03
Fruits and vegetables * visit	1.31	0.23

Supplementary Table 2. Multivariate regression with repeated measures for dietary AGE (kU/1000 kcal) by DM trial arm, study visit, and baseline characteristics

**Effect Modification:**	**F-statistic**	**P-value**
Fruits and vegetables * DM trial arm * visit	3.06	0.001
Whole grain quartiles, servings/day		
Whole grains * DM trial arm	1.51	0.21
Whole grains * visit	5.73	<0.0001
Whole grains * DM trial arm * visit	1.65	0.10
Red and processed meat quartiles, servings/day		
Red and processed meat * DM trial arm	0.25	0.86
Red and processed meat * visit	14.95	<0.0001
Red and processed meat * DM trial arm * visit	1.31	0.22
Total HEI-2015 score quartiles		
Total HEI-2015 score * DM trial arm	2.11	0.10
Total HEI-2015 score * visit	17.82	<0.0001
Total HEI-2015 score * DM trial arm * visit	5.15	<0.0001
AGE, Advanced Glycation End Product; DM, Dietary Modification; wk, week		

Supplementary Figures & Legends

Supplementary Figure 1. Dietary Advanced Glycation End Product (AGE) least squares means by dietary modification trial arm, study visit year at follow up and sociodemographic characteristics

Supplementary Figure 2. Dietary Advanced Glycation End Product (AGE) least squares means by dietary modification trial arm, study visit year at follow up and lifestyle behavioral characteristics

Supplementary Figure 3. Dietary Advanced Glycation End Product (AGE) least squares means by dietary modification trial arm, study visit year at follow up and dietary intake characteristics

Supplementary Figure 1. Dietary Advanced Glycation End Product (AGE) least squares means by dietary modification trial arm, study visit year at follow up and sociodemographic characteristics

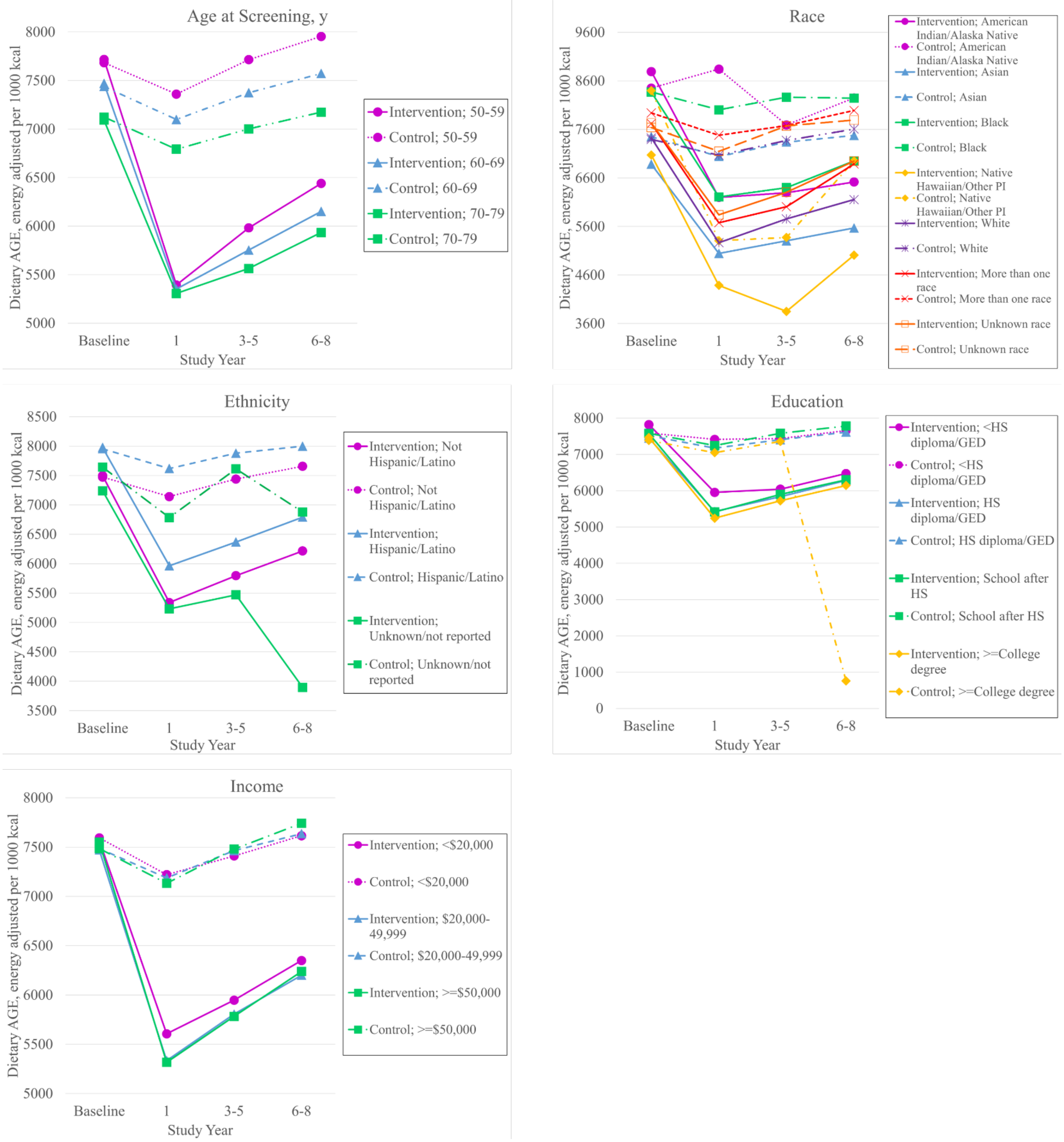

Supplementary Figure 2. Dietary Advanced Glycation End Product (AGE) least squares means by dietary modification trial arm, study visit year at follow up and lifestyle behavioral characteristics

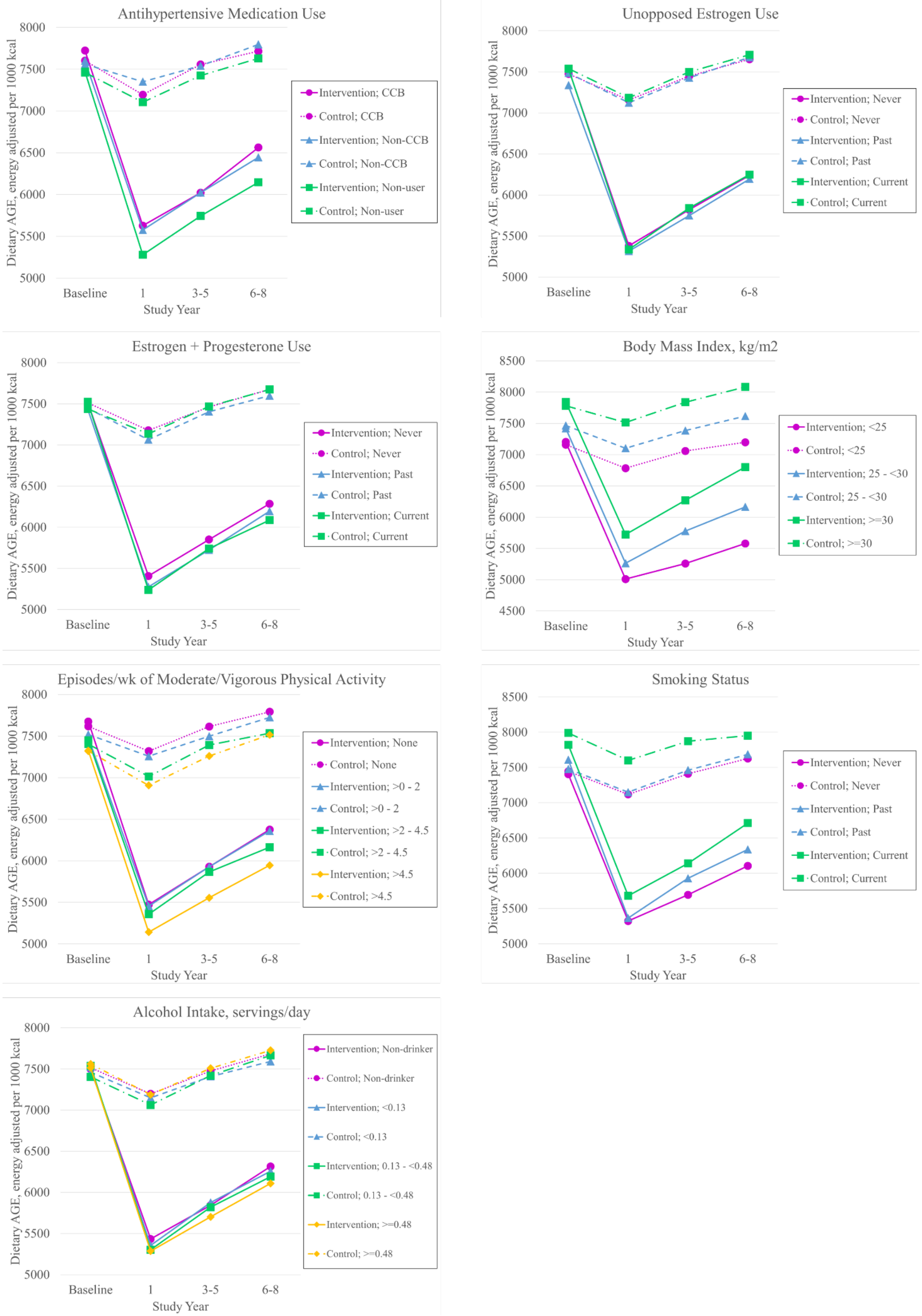

Supplementary Figure 3. Dietary Advanced Glycation End Product (AGE) least squares means by dietary modification trial arm, study visit year at follow up and dietary intake characteristics

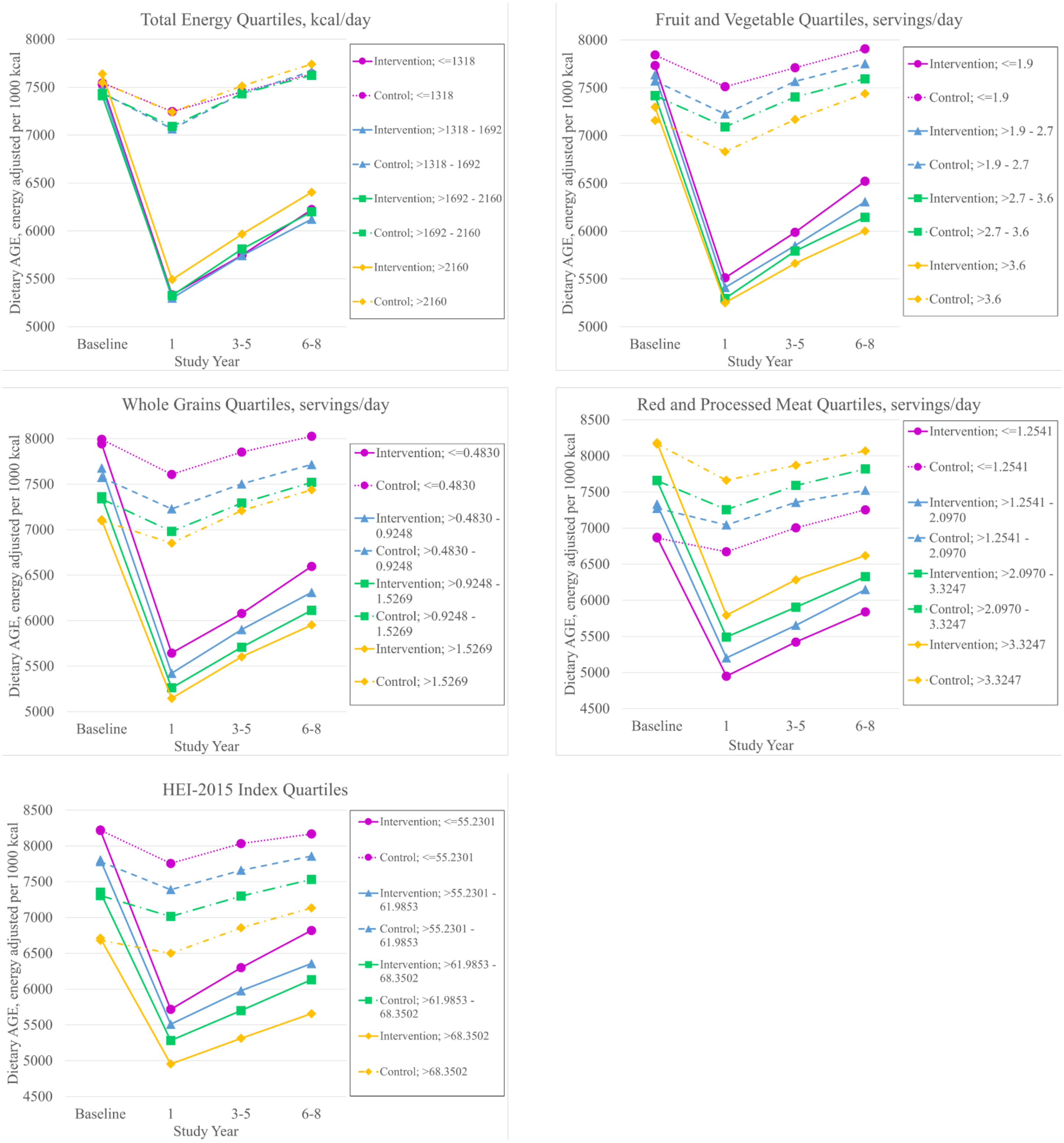

Supplement: Supplementary file 1 [file 41416_2026_3514_MOESM1_ESM.pdf]
